# Mediation of collisionless turbulent dissipation through cyclotron resonance

**DOI:** 10.1038/s41550-023-02186-4

**Published:** 2024-01-23

**Authors:** Trevor A. Bowen, Stuart D. Bale, Benjamin D. G. Chandran, Alexandros Chasapis, Christopher H. K. Chen, Thierry Dudok de Wit, Alfred Mallet, Romain Meyrand, Jonathan Squire

**Affiliations:** 1 Space Sciences Laboratory, University of California, Berkeley, Berkeley, CA USA; 2 Physics Department, University of California, Berkeley, Berkeley, CA USA; 3 Department of Physics & Astronomy, University of New Hampshire, Durham, NH USA; 4 Laboratory for Atmospheric and Space Physics, University of Colorado, Boulder, CO USA; 5 Department of Physics and Astronomy, Queen Mary University of London, London, UK; 6 LPC2E, CNRS and University of Orléans, Orléans, France; 7 International Space Science Institute, Bern, Switzerland; 8 Physics Department, University of Otago, Dunedin, New Zealand

**Keywords:** Solar physics, Astrophysical plasmas

## Abstract

The dissipation of turbulence in astrophysical systems is fundamental to energy transfer and heating in environments ranging from the solar wind and corona to accretion disks and the intracluster medium. Although turbulent dissipation is relatively well understood in fluid dynamics, astrophysical plasmas often exhibit exotic behaviour, arising from the lack of interparticle collisions, which complicates turbulent dissipation and heating in these systems. Recent observations by NASA’s Parker Solar Probe mission in the inner heliosphere have shed new light on the role of ion cyclotron resonance as a potential candidate for turbulent dissipation and plasma heating. Here, using in situ observations of turbulence and wave populations, we show that ion cyclotron waves provide a major pathway for dissipation and plasma heating in the solar wind. Our results support recent theoretical predictions of turbulence in the inner heliosphere, known as the helicity barrier, that suggest a role of cyclotron resonance in ion-scale dissipation. Taken together, these results provide important constraints for turbulent dissipation and acceleration efficiency in astrophysical plasmas.

## Abstract (web summary)

While turbulent dissipation is prevalent in astrophysics, the processes that convert turbulent energy into heat are often unclear. This study shows that plasma waves are fundamental to heating the solar wind and similar turbulent astrophysical systems.

## Main

Turbulence is an important means of energy transfer in astrophysical environments^1–8^. In hydrodynamics, turbulence manifests as the nonlinear shearing of structure in the velocity field (that is, eddies) into smaller scales, continuing until viscosity, which is mediated by interparticle collisions, dissipates the kinetic energy in the turbulence into thermal energy. Hydrodynamic turbulence is governed by the energy cascade rate through the dissipation of large-scale eddies as well as the fluid viscosity at microphysical scales. The independence of the energy cascade rate from the viscosity gives hydrodynamic turbulence a universal nature^9,10^. In contrast, astrophysical plasmas are often collisionless, and a variety of more exotic processes are responsible for dissipating turbulence into thermal energy.

The collisionless heating and acceleration of the solar wind and the solar corona remain poorly understood, yet fundamental, plasma processes with analogous dynamics occurring in many astrophysical systems^1–8^. As plasma leaves source regions on the solar surface, it undergoes continuous heating, resulting in a hot and tenuous upper atmosphere known as the solar corona. At coronal temperatures, the plasma cannot be confined by the Sun’s gravity and is accelerated into a supersonic solar wind that streams into the Solar System. Turbulent dissipation of energy stored in magnetic fields is a leading paradigm for explaining this process and is mostly collisionless in nature. Understanding these phenomena is a primary goal of NASA’s Parker Solar Probe (PSP) mission^11^.

Electromagnetic interactions in plasmas sustain a variety of waves that provide numerous pathways for the energy stored in turbulence to dissipate into thermal energy. At scales much larger than the ion gyroradius *ρ*_i_ = *v*_thi_/*Ω*_i_, where the ion temperature and mass, *T*_i_ and *m*_i_, define the ion thermal speed ${v}_\mathrm{thi}=\sqrt{2{T}_\mathrm{i}/{m}_\mathrm{i}}$ and the ion charge *q*_i_ sets the ion gyrofrequency *Ω*_i_ = *q*_i_*B*_0_/*m*_i_, magnetized turbulence is often polarized perpendicular to the mean background magnetic field **B**_0_, such that fluctuations in the velocity and magnetic field, δ**v** and δ**b**, approximately satisfy *δ***v**_⊥_ ≈ ±*δ***b**_⊥_ (ref. ^2^), where ${{{{\bf{\delta b}}}}}_{{{{\boldsymbol{\perp }}}}}={{{{\bf{\delta B}}}}}_{{{{\boldsymbol{\perp }}}}}/\sqrt{{\rho }_{0}{\mu }_{0}}$ and *ρ*_0_ is the average plasma mass density and μ_0_ is the permeability of free space. These qualities, common to Alfvén waves, lead to such turbulence being described as Alfvénic. The strength of the dominant Alfvén mode relative to subdominant fluctuations is referred to as the imbalance and can be quantified using the normalized cross-helicity ${\sigma }_{c}={2\langle \delta {{{{\bf{v}}}}}_{{{{\boldsymbol{\perp }}}}}\cdot \delta {{{{\bf{b}}}}}_{{{{\boldsymbol{\perp }}}}}\rangle }/({\langle \delta {{{{{\bf{v}}}}}_{{{{\boldsymbol{\perp }}}}}}^{2}\rangle +\langle \delta {{{{{\bf{b}}}}}_{{{{\boldsymbol{\perp }}}}}}^{2}\rangle })$, where values of *σ*_c_ ≈±1 indicate highly Alfvénic fluctuations.

The dissipation of Alfvénic fluctuations is negligible at large scales. Such fluctuations are often approximated using magnetohydrodynamics. Thus, energy must be transferred to scales comparable to *ρ*_i_ and the ion-inertial length ${d}_\mathrm{i}={\rho }_\mathrm{i}/\sqrt{{\beta }_\mathrm{i}}$, where ${\beta }_\mathrm{i}=2{n}_{0}{\mu }_{0}{T}_\mathrm{i}/{B}_{0}^{2}$ and *n*_0_ is the average number density. Near these ion-kinetic scales, Alfvén waves become dispersive and can be damped, which simultaneously heats the plasma and transfers energy into a turbulent cascade of kinetic Alfvén waves^12–15^.

In addition to kinetic Alfvén waves, plasmas sustain a variety of other electromagnetic waves that may play a role in plasma heating through wave–particle interactions^16–23^. Numerous populations of circularly polarized, electromagnetic waves at ion-kinetic scales have been documented by PSP^24^. These waves, which interact efficiently with particles through instabilities and can directly heat particles through resonant coupling to gyromotion, are one potential pathway to turbulent dissipation^5,6,16,21,25^.

Here, we show that signatures of turbulent dissipation are mediated by the presence of ion cyclotron waves (ICWs), which provides direct evidence for the role of cyclotron resonance in turbulent dissipation. The absence of ICWs is correlated with sub-ion kinetic turbulence that is populated with non-Gaussian, intermittent, fluctuations, which are likely indicative of dissipation through small-scale current sheets^23,26,27^ that have formed through kinetic Alfvén wave turbulence^12–14^. Our observations are consistent with a proposed helicity-barrier mechanism^28^, in which the conservation of energy and helicity prevent highly Alfvénic turbulence from cascading to sub-ion scales, resulting in the generation of ICWs that heat low-*β* magnetized plasmas^29^.

## Results

We study PSP observations for the full day of 27 September 2020^30,31^. The spacecraft was ∼30 *R*_⊙_ from the Sun.

### Turbulent properties

**Fig. 1:**
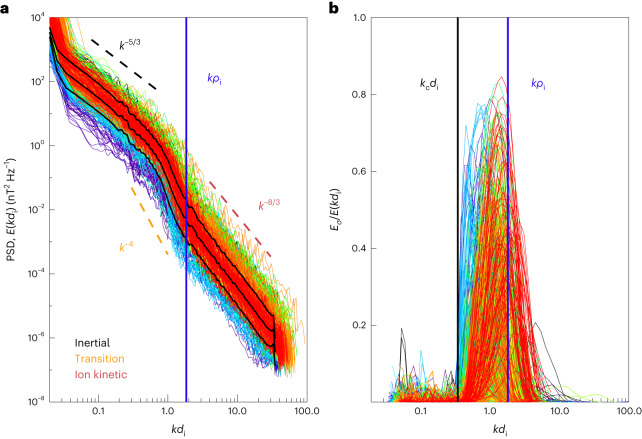
Turbulent power spectra measured by PSP. **a**, Observed trace of the PSD of magnetic field fluctuations for 747 intervals. Spectra are computed from measured time series using a fast Fourier transform. Frequencies are then normalized to the ion-inertial scale *kd*_i_ using the Taylor hypothesis. Spectra are coloured from blue to red by sequential occurrence in time on 27 September 2020. Dashed lines show various known spectral scalings: *k*^−5/3^, *k*^−8/3^ and *k*^−4^. **b**, Circularly polarized power normalized to trace power spectra computed from a wavelet transform. Frequencies are normalized to *kd*_i_ using the Taylor hypothesis. The solid vertical black line is an approximate lower-bound cutoff of the circularly polarized power *k*_c_*d*_i_ = 0.35. The blue line is the average position of the ion gyroscale *kρ*_i_.

Turbulent properties were studied using a set of 747 power spectral densities (PSDs) of trace magnetic field fluctuations *E*(*kd*_i_), where *k* is the wavenumber of the turbulent fluctuations (Methods), from 27 September 2020, as shown in Fig. 1a. The average ion-inertial length is 7.7 km, corresponding to a Doppler-shifted spacecraft frequency of 7.4 Hz with a standard deviation of 1.5 Hz. The average spectra and one standard deviation levels are shown as solid black lines. In the inertial range, the fluctuations are known to have an approximate power-law scaling with *k*^−*α*^. In agreement with previous results, we find *α*_I_ is 3/2 or 5/3 for *k*_⊥_ spectra, which is measured when the angle between the mean solar wind flow and **B**_0_, *θ*_BV_, is oblique, and *α*_I_ ≈ 2 for *k*_∥_ spectra, measured when *θ*_BV_ corresponds to a parallel or anti-parallel angle^32,33^. There is often a transition range at ion-kinetic scales with a very steep *α*_T_ ≈ 4 scaling^34–36^, whereas at sub-ion kinetic scales, a scaling with an approximate *α*_K_ ≈ −8/3 is observed^17,34,37,38^. Corresponding power-law scalings of −5/3, −4 and −8/3 are plotted on Fig. 1a to highlight these regions. Circular polarization is quantified using a Morlet wavelet transform (Methods). Figure 1b shows the circularly polarized power computed from a wavelet analysis for each of the 747 intervals. The power is normalized to the total power spectral density computed from the wavelet transform. Thus, a value of unity means that the power is entirely circularly polarized. We define a cutoff at *k*_c_*d*_i_ = 0.35, which is uniformly below the measured circularly polarized power in each interval. The blue line shows the average position of *kρ*_i_ with respect to *kd*_i_.

**Fig. 2:**
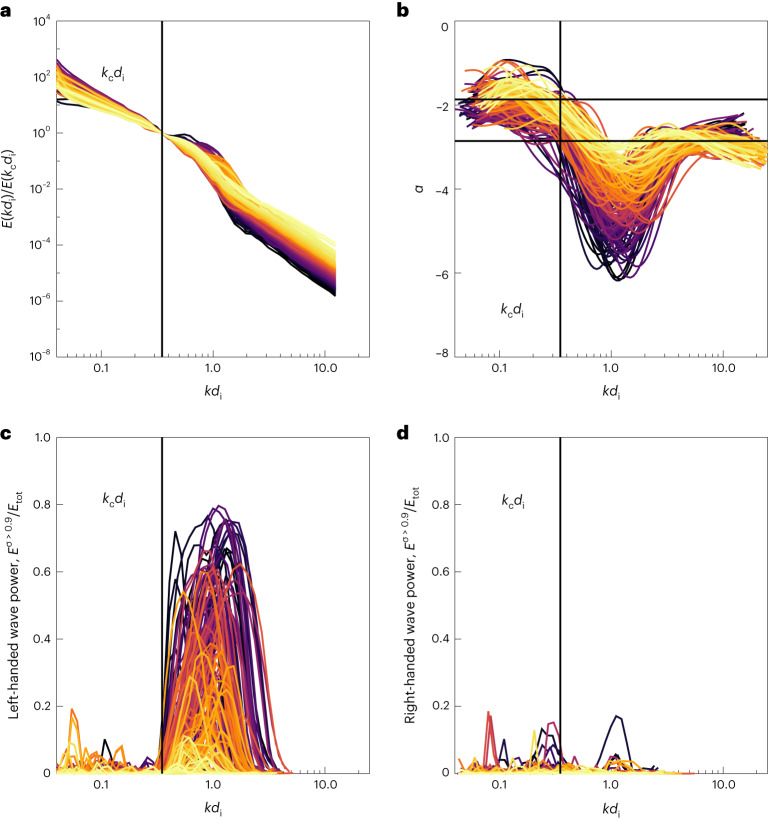
Spectral signatures of circularly polarized waves. **a**, Turbulent spectra from intervals with 15° < *θ*_BV_ < 25° normalized to the power at *k*_c_*d*_i_ = 0.35. Spectra are colour-coded by the difference in energy ${\tilde{E}}_\mathrm{TK}$ defined between *k*_c_*d*_i_ and *kd*_i_ = 10. Darker colours indicate a greater drop in power. **b**, Moving window spectral index for the 150 spectra. A break is observed near *k*_c_*d*_i_, which corresponds to the onset of circularly polarized power. Recovery to a power-law scaling of approximately −8/3 is recovered at sub-ion scales. Mean values of *α* in the inertial and kinetic ranges are shown as black horizontal lines. **c**, Circularly polarized power in left-handed modes normalized to the total power. **d**, Circularly polarized power in right-handed modes normalized to the total power.

Figure 2a shows spectra of the 150 intervals from Fig. 1 that satisfy 15° < *θ*_BV_ < 25°, which was imposed to control for effects due to anisotropy. This angle range was chosen to enable observation of ICWs^24^ while retaining some portions of the perpendicular spectra, which dominate the cascade^33^. Figure 2a shows power spectra with the power normalized to *E*(*k*_c_*d*_i_).

The ratio in power over the transition range ${\tilde{E}}_\mathrm{TK}=E\left(\right.k{d}_\mathrm{i}$ $=10\left.\right)/E({k}_\mathrm{c}{d}_\mathrm{i})$ (note that *kd*_i_ = 10 ≈ 29*k*_c_*d*_i_) was measured to quantify the drop in energy between transition and kinetic scales. Figure 2 is colour-coded by ${\tilde{E}}_\mathrm{TK}$, such that darker spectra have the greatest drop in power over the transition range, whereas the lightest colours correspond to the smallest drop in power. We similarly measured ${\tilde{E}}_\mathrm{IK}=E(k{d}_\mathrm{i}=10)/E(k{d}_\mathrm{i}=0.035)$ as the drop in energy spectra between inertial and kinetic scales. Figure 2b shows the moving window estimate of the spectral index *α* as a function of *kd*_i_. A clear steepening is observed near *kd*_i_ ≈ *k*_c_*d*_i_, with shallow spectra at *kd*_i_ < *k*_c_*d*_i_ and the recovery of a sub-ion kinetic-range scaling *α* ≈ 8/3 to 3 at *kd*_i_ ≫ *k*_c_*d*_i_ (ref. ^38^). The lowest frequency range has an average spectral index of around −2, consistent with known scalings of the spectra at 15° (ref. ^33^), although we note considerable variation in the low-frequency spectral index.

### Circularly polarized waves

The fraction of circularly polarized power *E*_*σ*_/*E*_⊥_ in each interval was determined using a Morlet wavelet transform (refs. ^24,39^, Methods). Figure 2c,d shows the normalized *E*_*σ*_/*E*_⊥_ from the wavelet transform for the left- and right-handed polarizations, respectively. Very little right-handed power is observed. The colour-coding shows that intervals with greater drops in power, a proxy for turbulent dissipation^36^, coincide with substantial left-handed circularly polarized power. We computed several correlations using the nonparametric Spearman ranked correlation coefficient *R*.

**Fig. 3:**
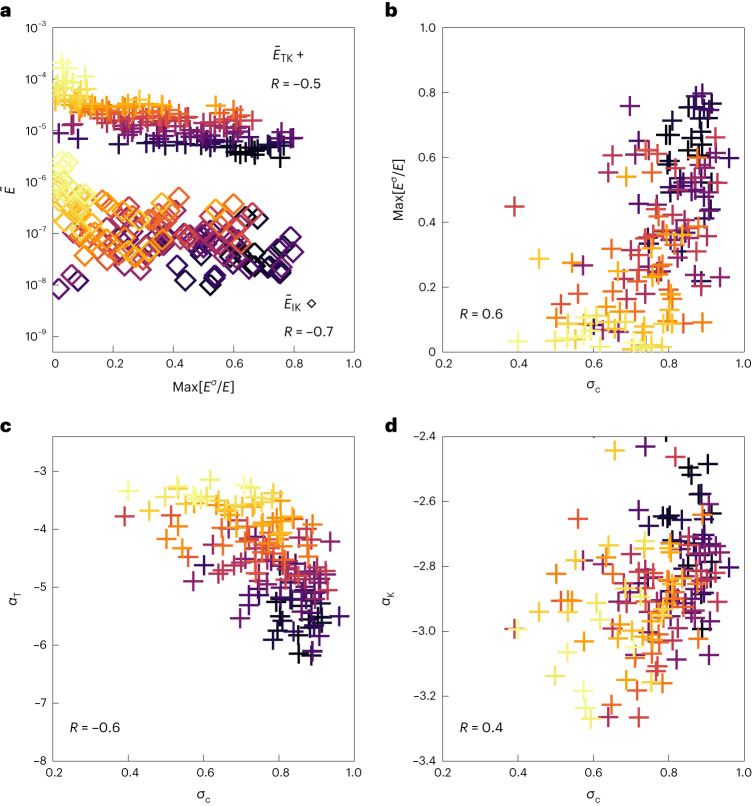
Correlating signatures of turbulence with waves. **a**, Scatter plot of drops in turbulent power (${\tilde{E}}_\mathrm{TK}$ and ${\tilde{E}}_\mathrm{IK}$) against the level of circular polarization. Plus signs (+) show ${\tilde{E}}_\mathrm{TK}$, the ratio in power between *kd*_i_ = 0.35 and *kd*_i_ = 10, corresponding to a drop in power between transition and sub-ion scales. Diamonds (◊) show ${\tilde{E}}_\mathrm{IK}$, the ratio between an inertial-range scale, *kd*_i_ = 0.035 and *kd*_i_ = 10. Ranked Spearman correlations of *R* = −0.5 and *R* = −0.7 are measured for ${\tilde{E}}_\mathrm{TK}$ and ${\tilde{E}}_\mathrm{IK}$, respectively. **b**, Level of circularly polarized power against cross-helicity. *R* = 0.6. **c**, Measured transition-range spectral index *α*_T_ plotted against the cross-helicity *σ*_c_. *R* = −0.6. **d**, Measured kinetic-scale spectral index *α*_k_ (computed at *kd*_i_ = 10) plotted against the cross-helicity *σ*_c_. *R* = 0.4. Colours for each data point are determined by ${\tilde{E}}_\mathrm{TK}$ and correspond to the spectra shown in Fig. 2.

**Fig. 4:**
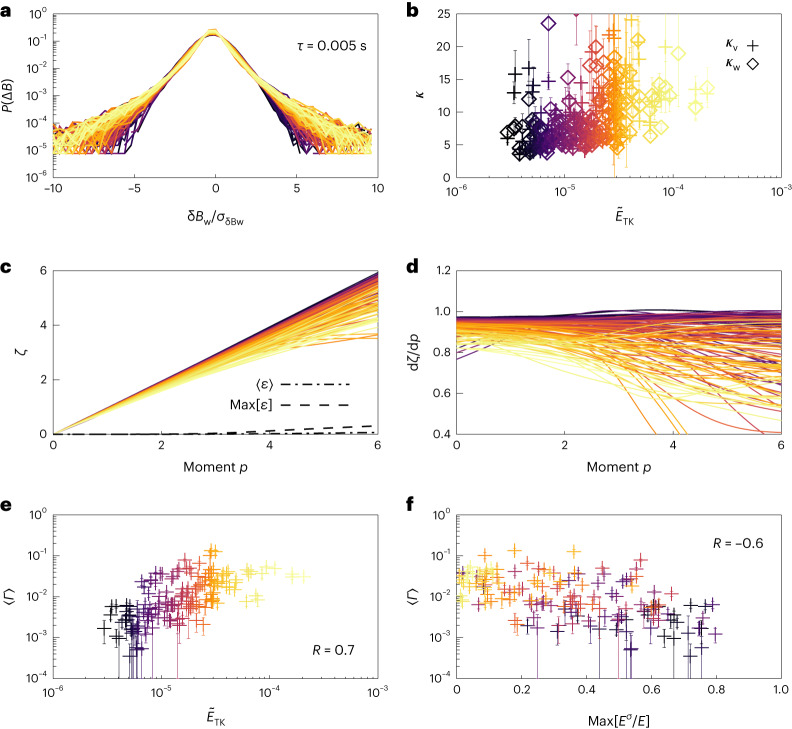
Effect of waves on kinetic-scale intermittent scaling. **a**, Distributions of increments at *τ* = 0.005 s for each interval in the SCM sensor coordinate $\hat{w}$ direction. Each distribution is normalized to its standard deviation. Colours correspond to the drop in energy ${\tilde{E}}_\mathrm{TK}$ shown in Fig. 2. **b**, Kurtosis of the fluctuations in the SCM $\hat{v}$ and SCM $\hat{w}$ directions (+ and ◊) at *τ* = 0.005 s plotted against ${\tilde{E}}_\mathrm{TK}$. Error bars on *κ* show the standard error for each interval obtained from bootstrapping each interval with an ensemble of 16 under-sampled distributions (Methods). **c**, Intermittent scaling for each interval is illustrated using the scaling exponent of the structure functions *ζ*(*p*). The standard error at each moment *p* for every interval is computed by bootstrapping (Methods). The dashed line shows the average standard error for the 150 studied intervals and the dotted-dashed line shows the maximum computed error. **d**, Derivative d*ζ*/d*p*. A linear *ζ*(*p*) (constant d*ζ*/d*p*) corresponds to non-intermittent fluctuations. **e**, Average curvature of sub-ion kinetic-range scaling exponent *Γ*_*ζ*_ plotted against the drop in spectra, ${\tilde{E}}_\mathrm{TK}$. **f**, Average curvature of sub-ion kinetic-range scaling exponent *Γ*_*ζ*_ plotted against the maximum circularly polarized power at ion-kinetic scales. Error bars on *Γ* show the standard error of the mean for each interval obtained from bootstrapping each interval with an ensemble of 16 under-sampled distributions (Methods).

Figure 3a shows the energy spectra ratios ${\tilde{E}}_\mathrm{TK}$ and ${\tilde{E}}_\mathrm{IK}$ plotted against the maximum circular polarization. Notably, strong correlations between the drop in power and presence of left-handed waves are present both for the drop over the transition and kinetic ranges, that is ${\tilde{E}}_\mathrm{TK}$ (*R* = −0.5), and for the inertial and the kinetic scales, ${\tilde{E}}_\mathrm{IK}$ (*R* = −0.7). Figure 3b shows the correlation between the maximum circularly polarized power computed over all *k*, $\max [{E}_{\sigma }/{E}_{\perp }]$, and the cross-helicity *σ*_c_. The correlation (*R* = 0.6) indicates that circularly polarized power is most often found in intervals with higher cross-helicity. Figure 3c shows that *α*_T_ is correlated (*R* = −0.6) to the level of cross-helicity at inertial scales. Figure 3d shows the correlation between the kinetic-range spectral index, computed between *kd*_i_ = 3.5 and *kd*_i_ = 10, and the cross-helicity. Higher cross-helicity is weakly correlated with flatter kinetic-scale spectral indices (*R* = 0.4). Together, the panels in Fig. 4 show that the turbulent dynamics at sub-ion scales is related to the presence of left-handed waves at ion scales and to the magnetohydrodynamic-scale cross-helicity. Furthermore, we show that high-cross-helicity states are preferentially associated with left-handed waves, which serve as a pathway to dissipation^25^.

Although the existence of ICWs may not inevitably result in the irreversible thermalization of turbulent energy, that is heat, we further analyse their connection to dissipation through studying their association with intermittency at sub-ion kinetic scales.

### Kinetic-scale intermittency

Figure 4 shows quantities relating to the kinetic-scale cascade. The distribution of increments $P({\Delta B}_j^{\tau })$ at *τ* = 0.005 s (Methods) for a single direction of the magnetic field measurements are shown in Fig. 4a. The colours again correspond to ${\tilde{E}}_\mathrm{TK}$, with darker colours associated with larger drops in power. Each $P({\Delta B}_j^{\tau })$ is normalized to the standard deviation of the distribution. There is a clear relationship between $P({\Delta B}_j^{\tau })$ and ${\tilde{E}}_\mathrm{TK}$, as lighter colours, that is smaller drops in power, have stronger non-Gaussian tails. This is further evidenced in Fig. 4b, which shows that larger drops in ${\tilde{E}}_\mathrm{TK}$ that are associated with the presence of ICWs (Fig. 2), have a lower kurtosis in the kinetic range.

Figure 4c shows the structure function scaling exponent *ζ*(*p*) (Methods) computed for each interval over the kinetic-scale range of 0.007 < *τ* < 0.027 s. The corresponding frequencies (18.3–97 Hz) are larger than those used in our wavelet analysis (0.36–17.5 Hz); thus, our analysis of intermittency is localized to a range of scales that are smaller than scales populated with waves. Error bars are not included in Fig. 4c for clarity, although the estimated levels of the mean and maximum errors in the scaling exponent are shown. The derivative d*ζ*/d*p* is shown in Fig. 4d. Darker colours are observed to be more self-similar and less intermittent, whereas the lighter colours show more notable deviations from self-similar scaling, indicating intermittency.

The nonlinearity in *ζ* is measured through the curvature of the scaling exponent *Γ*_*ζ*_. A larger *Γ*_*ζ*_ indicates less linear scaling in *ζ*(*p*), and thus greater intermittency. Figure 4e,f shows the average *Γ*_*ζ*_ from each interval plotted against ${\tilde{E}}_\mathrm{TK}$ and $\max [{E}_{\sigma }/{E}_{\perp }]$. Spearman ranked correlations of *R* = 0.7 and *R* = −0.6 were, respectively, obtained, indicating that kinetic-range intermittency is much stronger in intervals without strong wave activity.

## Discussion

These results show that left-handed waves interact strongly with turbulence at ion scales and consequently affect sub-ion kinetic-scale turbulence. Under the hypothesis that the solar wind consists of non-interacting turbulent fluctuations and circularly polarized waves, signatures of the turbulence deep in sub-ion scales should not correlate with the presence of ion-scale waves. The strong correlation between the decrease in the energy spectra with the level of left-handed circular polarization observed suggests that waves play an active role in dissipating energy from the turbulent cascade at these scales. Whether this process happens through direct absorption from the turbulence through a resonant interaction^16,17,21,40^ or whether the waves are generated following a primary mechanism (for example, oblique cyclotron resonance or stochastic heating^19,20,29^), the interaction between these waves and the turbulent cascade is well established by these observations.

Previous work has suggested that the variability in the transition-range spectral index may relate to properties of the inertial-range cascade^41,42^. Our results expand on these ideas by providing direct evidence that the presence of left-handed polarized ion-scale waves is highly correlated with the inertial-range cross-helicity. Although ICWs have long been suspected to play a role in heating collisionless environments^5,6,16,22,25^, our work provides strong evidence for the interaction of cyclotron waves with astrophysical turbulence and demonstrates an important pathway for turbulent dissipation and heating in the inner heliosphere and other *β* < 1 plasma environments.

Furthermore, we concretely demonstrate that ion-scale waves are correlated with signatures of sub-ion-scale turbulence. The sub-ion kinetic-scale kurtosis during intervals when ICWs are present is relatively low, suggesting that ion-scale dissipation may re-Gaussianize, that is randomize, the turbulent fluctuations^27,43^, which are known to be strongly intermittent in the inertial range^26^. The presence of ion-scale wave activity may inhibit the growth and production of intermittent structures, which are often associated with current sheets^23,26,27,35,44^ at sub-ion scales. We speculate that the steep drops across the transition range, which are associated with ICWs, may weaken the kinetic-scale nonlinearities^45^, such that nonlinear interactions no longer produce strongly intermittent fluctuations. This suggests that intermittent scalings observed at kinetic scales^23,27,35,46^ are related to the available pathways for ion-scale dissipation and heating.

Although the exact means of dissipating small-scale coherent structures remains largely unconstrained^23,43^, our results support their importance in collisionless plasma heating^23,37,38,47^. The mediation of energy transfer by ion-scale waves suggests that the fraction of turbulent energy deposited in the ions through cyclotron-resonant interactions versus that deposited in electrons through kinetic-scale turbulence^8,38,45,48^ depends on the large-scale cross-helicity of the turbulence. The dependence of dissipative mechanisms on the large-scale geometry of the turbulent fluctuations is a striking contrast to hydrodynamic turbulence in which turbulence is dissipated only through the viscosity.

Our results support recent suggestions that turbulence in high-cross-helicity states may not be able to physically transfer the majority of turbulent energy to kinetic scales due to the need for the simultaneous conservation of both helicity and energy^28,29^. This helicity barrier^28^ implies that the build-up of energy in the inertial range results in the growth of fluctuations with small parallel scales, which damps the turbulence through cyclotron resonance^49^. Regions with high cross-helicity are able to transfer only a small fraction of the turbulent energy flux to sub-ion scales, implying that there is a larger spectral drop through the transition range and smaller-amplitude sub-ion turbulence. Sufficiently small-amplitude sub-ion turbulence may render the sub-ion-scale turbulence weak, which may explain our observed correlations among sub-ion intermittency, the large-scale cross-helicity and pronounced transition-range steepening.

Our observation that turbulent dissipation depends on the large-scale cross-helicity suggests a non-universal nature of solar wind turbulence, which likely has important implications for the acceleration and global structure of the corona and solar wind. In particular, the observation that imbalanced turbulence dissipates into ion heat through ion cyclotron fluctuations suggests that turbulence in fast-wind streams, which are often imbalanced, should predominantly heat ions. Conversely, our observation of stronger, more intermittent sub-ion turbulence for less imbalanced intervals suggests that turbulence in lower-Alfvénicity slow-wind streams should predominantly heat electrons. Because of the electrons’ higher thermal velocities, a quantity of energy deposited into ion heat at distances far from the Sun is especially efficient at accelerating the plasma to high speeds, as opposed to simply heating it up^50^. Thus, the large-scale turbulence imbalance, by controlling the plasma’s dissipative mechanisms, could directly influence the acceleration efficiency of a parcel of plasma. Such a mechanism could potentially combine with other well-known processes that link turbulence properties and acceleration^51^ to control the solar wind’s acceleration and properties in different regions.

These observations have important ramifications for understanding energy transfer in diverse astrophysical systems such as the intracluster medium^7^ and accretion disks^4^. These systems are highly important to our understanding of the Universe but can be observed only through their radiative signatures. Although studies of thermodynamics and particle heating have largely been considered as functions of *T*_p_/*T*_e_ and *β* (refs. ^4,52^), our results highlight the importance of understanding the turbulent properties, for example the cross-helicity, that may affect dissipation and heating processes in these environments. Further work connecting these in situ observations of heating in heliospheric plasmas with astrophysical environments is likely to benefit our understanding of processes occurring broadly in the Universe.

## Methods

We investigated PSP observations^11^ for the full day of 27 September 2020. Measurements of the local plasma conditions are made with the PSP Solar Wind Electron Alphas and Proton experiment^31^. We measured a mean speed for this stream of 〈*V*_sw_〉 = 355 km s^−1^. Moreover, 〈*T*_i_〉 = 55 eV, average magnetic field magnitude 〈*B*〉 = 255 nT, 〈*n*_e_〉 = 1,200 cm^−3^ and the average 〈*β*_i_〉 = 0.43.

We use merged search-coil (SCM) and fluxgate magnetometer data from the PSP FIELDS instrument suite^30,53^ to enable observations of the turbulent cascade from the inertial to the kinetic ranges^36^. Wave polarization was studied with the fluxgate magnetometers, as the search-coil and merged data have only two functioning components^54^. The total electron density was obtained from the FIELDS quasithermal noise measurements^55^. Data were separated into 747 intervals of approximately 224 s (131,072 samples at ∼586 samples per second). Each individual interval overlapped 50% with neighbouring intervals to increase the size of the statistical ensemble. The turbulent spectra were determined through the trace PSD *E*(*f*) of the magnetic field through a Fourier transform. The Taylor hypothesis was used to convert spacecraft frequency *f* to wavenumber *k* as *f* = 2π*kv*_sw_, as the solar wind speed was higher than the Alfvén speed. Each spectrum was interpolated onto 56 logarithmically spaced frequencies ranging from ∼0.06*f*_di_ to ∼12*f*_di_, where *f*_di_ corresponds to *kd*_i_ = 1. The maximum scale 12.4*kd*_i_ was chosen to avoid the instrumental noise floor at higher frequencies^53^. Below approximately 0.03*kd*_i_, an artefact was introduced due to the logarithmic interpolation of the PSD, for example, see Fig. 1a. We did not include these scales in our analysis, and the artefact did not affect our results. The variation in *α* with wavenumber was studied by measuring the local slope of the logarithmically spaced PSD in a 13-point window centred at each frequency. The transition-range spectral index *α*_T_ was taken as the minimum of the local slope in the moving window. The kinetic range *α*_K_ index is the slope computed at 10*kd*_i_. The inertial range *α*_I_ index is the slope computed at 10*kd*_i_.

A 36-scale Morlet wavelet transform, with a response between 0.4 and 18 Hz, was used to identify circularly polarized waves:

1 $$\tilde{B}(s,\tau )=\mathop{\sum }\limits_{i = 0}^{N-1}\psi \left(\frac{{t}_{i}-\tau }{s}\right)B({t}_{i}).$$

The complex-valued wavelet transform was rotated into a field-aligned coordinate system parallel to the local background field: ${\tilde{\bf{B}}}_\mathrm{FAC}=$ $({\tilde{B}}_{\perp 1},{\tilde{B}}_{\perp 2},{\tilde{B}}_{\parallel })$. We studied signatures of circular polarization using ${\sigma }_{B}(\;f,t)=-2\,{{\mbox{Im}}}\,({\tilde{B}}_{\perp 1}{\tilde{B}}_{\perp 2}^{* })/({\tilde{B}}_{\perp 1}^{2}+{\tilde{B}}_{\perp 2}^{2})$, with left- and right-handed waves corresponding to positive or negative helicity, respectively. Waves were identified when ∣*σ*_B_∣ > 0.9 (refs. ^24,36^). Negative values of *σ*_B_ correspond to a left-handed rotation of the field, whereas positive values of *σ*_B_ correspond to right-handed polarization. The fraction of circularly polarized power was determined by filtering wavelet coefficients with ∣*σ*_B_∣ > 0.9 and normalizing the polarized power *E*_∣*σ*∣>0.9_(*f*) to the total observed power ${E}_{\perp }(\,f\,)=| {\tilde{B}}_{\perp 1}^{2}+{\tilde{B}}_{\perp 2}^{2}|$.

When the angle between the solar wind flow and the mean magnetic field *θ*_BV_ is sufficiently oblique, observing quasi-parallel waves is complicated, as the polarization plane of parallel-propagating circularly polarized waves is not aligned with the flow over the spacecraft^24^. Additionally, solar wind turbulence is anisotropic^33^, such that observed perpendicular turbulent fluctuations can dominate over parallel-propagating ICWs at oblique *θ*_BV_. To control for these observational effects, we considered only intervals with 15° < 〈*θ*_BV_〉 < 25° to control for anisotropy^33^ while capturing a sufficient ensemble of intervals with and without wave signatures. Our results are robust to varying the range of *θ*_BV_, even when a large range, for example 0° < *θ*_BV_ < 40°, is studied. However, the anisotropy associated with the large range of *θ*_BV_ may affect the results^24^. Although single-point measurements pose inherent limitations, selecting only intervals 15° < *θ*_BV_ < 25° enables us to observe the scaling properties of the perpendicular turbulent cascade alongside quasi-parallel waves. Although our inability to study the full three-dimensional turbulent cascade in the presence of ion-scale waves is unfortunate, our observations of the quasi-parallel spectra provide crucial evidence for the mediation of turbulent dissipation through cyclotron resonance.

To relate the presence of waves to observed signatures of kinetic-scale turbulence, we computed magnetic field increments,

2 $$\Delta {B}_j^{\tau }={B}_j(t)-{B}_j(t+\tau ),$$

for vector components indexed by *j* and lag *τ* ranging from ∼0.005 to 7 s (∼0.1–100 Hz). The probability distribution of increments at a given *τ*, denoted as $P({\Delta B}_j^{\tau })$, characterizes signatures in the fluctuations that relate to turbulent dissipation, for example non-Gaussianity and intermittency^26,43^.

Structure functions of the increments of order *p* are constructed as ${S}_j^{p}(\tau )=\langle | \Delta {B}_j^{\tau }{| }^{p}\rangle$, where 〈.〉 indicates an average over each 224 s interval. Moments of $P({\Delta B}_j^{\tau })$ are approximately accurate up to $p < \log N-1$ (ref. ^56^). As *N* = 131,072, moments with *p* ≤ 4 are likely well measured in each interval. Signatures of turbulent heating and dissipation are known to correlate with the non-Gaussianity of the fluctuations^23,26^. Deviations from Gaussianity are often measured using the kurtosis of the fluctuations $\kappa {(\tau )}_{\Delta {B}_j}={S}_j^{4}(\tau )/{S}_j^{2}{(\tau )}^{2}$. A Gaussian distribution has *κ* = 3, with larger values corresponding to heavy-tailed distributions that indicate that there is a non-Gaussian structure^23,26^. Intermittency in turbulence is usually defined as increasing non-Gaussianity in $P({\Delta B}_j^{\tau })$ as the scale size decreases.

Intermittency is also evident in the scaling of moments of the $P({\Delta B}_j^{\tau })$ distribution over various *τ* (refs. ^26,27,35,44^). Non-intermittent scalings of $P({\Delta B}_j^{\tau })$ correspond to self-similarity at each *τ* with

3 $$P\left(\Delta {B}_j^{\tau }\right)={\tau }^{-H}f\left(\Delta {B}_{j}^{\tau }/{\tau }^{H}\right),$$

where *f*(*x*) is an arbitrary function and *H* is some number. The prefactor *τ*^−*H*^ allows for normalization:

4 $$1=\int{}P\left(\Delta {B}_j^{\tau }\right)\,\mathrm{d}\Delta B,$$

at each *τ*. Self-similar scaling of $P({\Delta B}_j^{\tau })$ results in structure functions that scale as ${S}_{j}^{p}(\tau )\propto {\tau }^{\;\zeta\, (\;p)}$ with *ζ*(*p*) = *Hp* (refs. ^26,35^). In general, if $P({\Delta B}_j^{\tau })$ is not self-similar according to equation (3), then ${S}_{j}^{p}(\tau )\propto {\tau }^{\zeta (p)}$, where the increasing non-Gaussian structure in intermittent fluctuations results in a nonlinear *ζ*(*p*) (ref. ^26^). We tested for intermittency in kinetic-scale turbulence by measuring *ζ*(*p*) in 20 Hz < *f* < 100 Hz, which corresponds to sub-ion scales. A constant d*ζ*/d*p* indicates that the kinetic-scale fluctuations show self-similarity whereas a variable d*ζ*/d*p* indicates intermittency with multi-fractal properties.

We computed the curvature in *ζ* for each interval ${\varGamma }_{\zeta }=\frac{| {\zeta }^{\,{\prime\prime} }(p)| }{{\left(1+{\zeta }^{\,{\prime} }{(p)}^{2}\right)}^{3/2}}$ to attain a single number for the nonlinearity of the scaling exponents, which could be correlated against other observable parameters. We measured and reported the average *Γ* over *p* in each interval. The study was repeated using the maximum *Γ*_*ζ*_ in each interval, although no notable variation in the results was obtained.

Errors on each of these quantities were approximated by bootstrapping methods. We generated a list of 10,000 random indices from each *N* = 131,072 sample interval. From this sub-sampled interval, we estimated the kurtosis *ζ*(*p*) and *Γ*_*ζ*_(*p*) using the above methods. This process was repeated 16 times for each interval to give an ensemble of estimated values for *κ*, *ζ*(*p*) and *Γ*_*ζ*_(*p*). The error of the mean estimates are reported as the standard error of this distribution. For errors on *ζ*(*p*), we did not consider the error on each *p* for interval individually but considered both the average and maximum measured error for each *p*, as shown in Fig. 4c. We found that error on *ζ*(*p*) increased with *p*, which is consistent with the behaviour expected due to finite-sample effects. Moments up to *p* ≈ 3 should be accurately estimated^56^. We found that the average standard error 〈*ϵ*_*ζ*_〉 at *p* = 3 was 0.008 and the maximum measured standard error was $\max [{\epsilon }_{\zeta }]=0.05$. At *p* = 6, the average standard error 〈*ϵ*_*ζ*_〉 was 0.06 and the maximum measured standard error was $\max [{\epsilon }_{\zeta }]=0.36$. These errors are relatively small as *ϵ*_*ζ*_/*ζ* < ∼10%. This same method was used to determine the standard error of the derivative d*ζ*/d*p*, ${\epsilon }_{\zeta }^{{\prime} }$, which is of order 1%. Estimating the error of the curvature *Γ*_*ζ*_ was similarly performed using the standard error of the mean determined from the standard deviation of the 16-element ensemble.

## Data Availability

PSP data are publicly available at NASA Space Physics Data Facility (SPDF) https://cdaweb.gsfc.nasa.gov/. FIELDS data are also hosted at https://sprg.ssl.berkeley.edu/data/psp/data/sci/fields/.
